# A systematic review of salivary gland hypofunction and/or xerostomia induced by non-surgical cancer therapies: prevention strategies

**DOI:** 10.1007/s00520-024-09113-x

**Published:** 2025-01-10

**Authors:** Valeria Mercadante, Derek K. Smith, Ragda Abdalla-Aslan, Ana Andabak-Rogulj, Michael T. Brennan, Graziella Chagas Jaguar, Haley Clark, Eduardo Rodrigues Fregnani, Luiz Alcino Gueiros, Allan Hovan, Seema Kurup, Alexa M. G. A. Laheij, Charlotte Duch Lynggaard, Joel J. Napeñas, Douglas E. Peterson, Sharon Elad, Stephanie Van Leeuwen, Arjan Vissink, Jonn Wu, Deborah P. Saunders, Siri Beier Jensen

**Affiliations:** 1 Eastman Dental Institute, University College London, London, England; 2 Vanderbilt University, Nashville, TN USA; 3 Bruce Rappaport Faculty of Medicine, Technion-Israel Institute of Technology, Haifa, Israel; 4 Department of Oral and Maxillofacial Surgery, Rambam Health Care Campus, Haifa, Israel; 5 Department of Oral Medicine, University of Zagreb School of Dental Medicine, Zagreb, Croatia; 6 Department of Oral Medicine/Oral & Maxillofacial Surgery, Atrium Health Carolinas Medical Center, Charlotte, NC USA; 7 Stomatology Department - A.C. Camargo Cancer Center, Sao Paulo, Brazil; 8 Department of Medical Physics, BC Cancer, Surrey, BC Canada; 9 Centro de Oncologia Molecular, Hospital Sírio-Libanês, São Paulo, Brazil; 10 Departamento de Clínica E Odontologia Preventiva, Universidade Federal de Pernambuco, Recife, Brazil; 11 Oral Oncology/Dentistry, British Columbia Cancer Agency-Vancouver Centre, Vancouver, BC Canada; 12 Department of Oral & Maxillofacial Diagnostic Sciences, School of Dental Medicine, UConn Health, Farmington, CT USA; 13 Department of Oral Medicine, Academic Centre for Dentistry Amsterdam, University of Amsterdam and VU University, Amsterdam, The Netherlands; 14 Department of Oral and Maxillofacial Surgery, Amsterdam UMC, University of Amsterdam, Amsterdam, The Netherlands; 15 Department of Otorhinolaryngology, Head and Neck Surgery and Audiology, Rigshospitalet, Copenhagen University Hospital, Copenhagen, Denmark; 16 Department of Oral Medicine/Oral & Maxillofacial Surgery, Atrium Health Carolinas Medical Center, Charlotte, NC USA; 17 School of Dental Medicine and Neag Comprehensive Cancer Center, UConn Health, Farmington, CT USA; 18 Eastman Institute for Oral Health, University of Rochester Medical Center, Rochester, NY USA; 19 Department of Dentistry, Radboud University Medical Center, Nijmegen, The Netherlands; 20 Department of Oral & Maxillofacial Surgery, University Medical Center Groningen, University of Groningen, Groningen, The Netherlands; 21 British Columbia Cancer Agency, University of British Columbia, Vancouver, Canada; 22 North East Cancer Center, Health Sciences North, Northern Ontario School of Medicine, Sudbury, ON Canada; 23 Department of Dentistry and Oral Health, Faculty of Health, Aarhus University, Aarhus, Denmark

**Keywords:** Salivary gland hypofunction, Xerostomia, Radiotherapy, Chemotherapy, Checkpoint inhibitors

## Abstract

**Purpose:**

This systematic review aimed to assess the updated literature for the prevention of salivary gland hypofunction and xerostomia induced by non-surgical cancer therapies.

**Methods:**

Electronic databases of MEDLINE/PubMed, EMBASE, and Cochrane Library were searched for randomized controlled trials (RCT) that investigated interventions to prevent salivary gland hypofunction and/or xerostomia. Literature search began from the 2010 systematic review publications from the Multinational Association of Supportive Care in Cancer/International Society of Oral Oncology (MASCC/ISOO) up to February 2024. Two independent reviewers extracted information regarding study design, study population, cancer treatment modality, interventions, outcome measures, methods, results, risk of bias (RoB version 2), and conclusions for each article.

**Results:**

A total of 51 publications addressing preventive interventions were included. Eight RCTs on tissue-sparing radiation modalities were included showing significant lower prevalence of xerostomia, with unclear effect on salivary gland hypofunction. Three RCTs on preventive acupuncture showed reduced prevalence of xerostomia but not of salivary gland hypofunction. Two RCTs on muscarinic agonist stimulation with bethanechol suggested a preventive effect on saliva flow rate and xerostomia in patients undergoing head and neck radiation or radioactive iodine therapy. Two studies on submandibular gland transfer showed higher salivary flow rates compared to pilocarpine and lower prevalence of xerostomia compared to no active intervention. There is insufficient evidence on the effectiveness of vitamin E, amifostine, photobiomodulation, and miscellaneous preventive interventions.

**Conclusion:**

This systematic review continues to support the potential of tissue-sparing tecniques and intensity-modulated radiation therapy (IMRT) to preserve salivary gland function in patients with head and neck cancer, with limited evidence on other preventive strategies, including acupuncture and bethanecol. Preventive focus should be on optimized and new approaches developed to further reduce radiation dose to the parotid, the submandibular, and minor salivary glands. As these glands are major contributors to moistening of the oral cavity, limiting the radiation dose to the salivary glands through various modalities has demonstrated reduction in prevalence and severity of salivary gland hypofunction and xerostomia. There remains no evidence on preventive approaches for checkpoint inhibitors and other biologicals due to the lack of RCTs.

**Supplementary Information:**

The online version contains supplementary material available at 10.1007/s00520-024-09113-x.

## Introduction

Saliva contributes a crucial role in oral health, including preserving integrity of dentition, periodontium, and mucosa, as well as mechanical cleansing of the oral cavity. Saliva’s antimicrobial activity aids in prevention of oral infections and also plays an important part in taste, formation of food bolus, facilitation of mastication, swallowing and speech, as well as lubrication of oropharyngeal and upper esophageal mucosa [12]. Saliva is produced from the major salivary glands (parotid, submandibular, and sublingual) which account for 90% of the saliva production, and the minor salivary glands which account for the remaining 10%. The submandibular glands, comprised of both serous and mucous acinar cells, produce approximately two-thirds of the saliva under resting conditions. Upon mechanical stimulation, the serous parotid glands will produce a watery and protein-rich fluid that accounts for about half of the total volume of saliva, the remainder mainly being submandibular secretion. Although the minor salivary glands are responsible for producing 10% of the total volume of saliva during resting conditions, they play a significant role in lubricating the mucosa [12].

Hyposalivation, characterized by low saliva secretion, is commonly defined as a resting whole saliva flow rate of ≤ 0.1 ml/min and/or a stimulated whole saliva flow rate of ≤ 0.7 ml/min [59]. The sensation of oral dryness may occur when a person’s normal unstimulated whole saliva flow rate is reduced by about 45–50% [35], although the two events are not always related. Due to the functional importance of saliva, a reduced salivary flow rate can lead to an increased risk of developing oral infections [27] and carious destruction of teeth [11], oral mucosal discomfort and pain, hampered oral functioning, and a worsened nutritional state [43]. As a consequence, patients with salivary gland hypofunction usually are restricted in their daily activities, have a poorer general well-being, and are limited in their social interactions [55].

Following head and neck cancer treatment, salivary gland hypofunction (objective evidence of low salivary flow rate) and xerostomia (subjective feeling of dry mouth) are predictable long-term complications [39]. This is particularly relevant following radiotherapy involving exposure of the major and minor salivary glands [5]. In contrast, salivary gland hypofunction following chemotherapy as well as other cancer treatments such as radioactive iodine treatment and total body irradiation/hematopoietic stem cell transplantation (HSCT), are less prevalent, often temporary and, where present, less severe [26]. In patients treated with targeted therapies, e.g., immune checkpoint inhibitors, a xerostomia prevalence of 0.4–7% has been reported with or without associated dry eyes [65]. Further studies are warranted to explore this correlation as reported prevalence might be counfonded by polypharmacy and dehydration.

This systematic review represents a search and evaluation of the literature appearing since the previous MASCC/ISOO systematic review conducted in 2010 (MASCC/ISOO) [29] on the prevention of salivary gland hypofunction and xerostomia induced by non-surgical cancer therapies, including literature on the newer checkpoint inhibitors and other biologicals which have now become an integral part of cancer therapies. Data collected in this systematic review of the literature also served as the basis for the ISOO/MASCC/ASCO clinical practice guideline: Salivary Gland Hypofunction and/or Xerostomia Induced by Nonsurgical Cancer Therapies [40].

## Systematic review methodology

### Search strategy and criteria for selecting studies

This systematic review was conducted by the Oral Care Study Group of ISOO/MASCC. PubMed, EMBASE, and Cochrane Library were searched for randomized controlled trials (RCTs) published in the English language that investigated interventions aimed at preventing salivary gland hypofunction and/or xerostomia induced by non-surgical cancer therapies. The literature search interval was January 1, 2009, through February 16, 2024, using combinations of the MeSH terms (Supplementary Document). The search results were imported into a computerized database (Endnote Version X9). The search results from each of the electronic databases of MEDLINE/PubMed and EMBASE were combined, and duplicate publications were eliminated. Articles were selected for inclusion in the systematic review of the evidence based on the following criteria:

- Population: Adult patients with cancer who were scheduled to receive non-surgical cancer therapy.
- Cancer types included: Head and neck cancer (radiation therapy in the head and neck region, chemotherapy, or chemoradiotherapy), hematologic malignancies (HSCT, systemic chemotherapy, and total body irradiation as cancer treatment and conditioning regimens), thyroid cancer (radioactive iodine), other solid cancer (systemic cancer chemotherapy), all cancers treated by biologic cancer therapy including targeted therapies.
- Fully published RCTs.

Articles were excluded from the systematic review if they were meeting abstracts not subsequently published in peer-reviewed journals, editorials, commentaries, letters, news articles, case reports, and narrative reviews; or published in a non-English language. RCTs of salivary gland hypofunction were included when objective measurement of salivary gland function was performed. The abstract of each article was reviewed by the Salivary Gland Hypofunction and Xerostomia Co-Section Heads (V.M. and S.B.J.) from the Oral Care Study Group, MASCC/ISOO. Irrelevant citations were removed according to the abovementioned criteria mentioned above (publication types) creating a preliminary set of potentially relevant publications. The selected full-text articles were distributed to the reviewer team along with an evaluation form customized in a Research Electronic Data Capture (REDCap®) database for reviewing salivary gland hypofunction and xerostomia data. All reviewers completed a calibration exercise before data collection, and feedback was submitted by e-mail correspondence. For each publication, two independent reviewers extracted information regarding the study design, study population, interventions, outcome measures, methods, results, risk of bias, and conclusions. The Cochrane risk-of-bias tool for randomized trials (RoB version 2) criteria was used to assess elements of quality related to study design, methodology, and the risk of bias in RCT. Results of salivary flow rates and prevalence and severity of xerostomia were extracted. The evaluation results were compared and re-evaluated until consensus was reached between two reviewers. A third reviewer was consulted to reach consensus. The reviewers were part of the Oral Care Study Group of ISOO/MASCC and included experts in oral medicine, oral pathology, clinical oral physiology, oral oncology, oncology nursing, radiation oncology, oral immunology, pediatric dentistry, oral and maxillofacial surgery, palliative oncology, periodontology, epidemiology, and biostatistics.

## Results

### Description of included studies

**Fig. 1:**
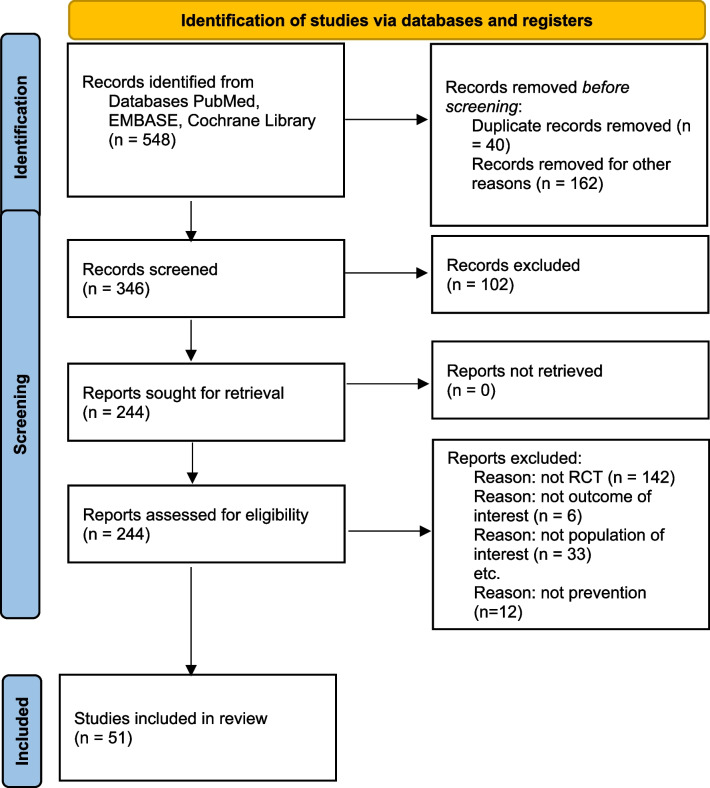
PRISMA (Preferred Items for Systematic Review and Meta-Analysis) flow diagram of the systematic review

The electronic searches identified over 500 titles and abstracts, from which 239 potentially relevant publications were selected according to the defined criteria. After the review group examined the abstracts and full-text articles, 51 articles were considered eligible (Fig. 1 – PRISMA flow diagram of the systematic review). Description of included studies can be found in Table 1.

**Table 1:** List of included studies

Randomized controlled trials	Number of participants	Intervention	Outcome measures		Key findings	Risk of bias
Tissue-sparing radiation techniques, IMRT						
Ghosh-Laskar S, Yathiraj PH, Dutta D, Rangarajan V, Purandare N, Gupta T, Budrukkar A, Murthy V, Kannan S, & Agarwal JP. (2016). Prospective randomized controlled trial to compare 3-dimensional conformal radiotherapy to intensity-modulated radiotherapy in head and neck squamous cell carcinoma: Long-term results. Head & Neck, 38 Suppl 1, E1481-1487	59	Experimental arm: IMRT Control arm: 3D-RT	Xerostomia: RTOG Salivary gland hypofunction: NA		At 2 years and 5 years the prevalence of grade 2 or worse xerostomia was significantly lower in the experimental arm (0% both time points) compared to the control arm (27.7% and 16.7% respectively)	Some concerns: randomization process, deviations from the intended interventions, missing outcome data and measurement of the outcome
Gupta T, Agarwal J, Jain S, Phurailatpam R, Kannan S, Ghosh-Laskar S, Murthy V, Budrukkar A, Dinshaw K, Prabhash K, Chaturvedi P, & D’Cruz A. (2012). Three-dimensional conformal radiotherapy (3D-CRT) versus intensity-modulated radiation therapy (IMRT) in squamous cell carcinoma of the head and neck: a randomized controlled trial. Radiotherapy and Oncology: Journal of the European Society for Therapeutic Radiology and Oncology, 104(3), 343–348	60	Experimental arm: IMRT Control arm: 3D-CRT	Xerostomia: RTOG Salivary gland hypofunction: NA		At 4–6 weeks after treatment completion, the prevalence of grade 2 or worse xerostomia was significantly lower in the experimental arm (63%) compared to the control arm (83%)	High
Kang M, Wang F, Liao X, Zhou P, & Wang R. (2018). Intensity-modulated radiotherapy combined with endostar has similar efficacy but weaker acute adverse reactions than IMRT combined with chemotherapy in the treatment of locally advanced nasopharyngeal carcinoma. Medicine, 97(25):e11118	23	Experimental arm: IMRT + recombinant human endostatin (angiogenesis inhibitor) Control arm: IMRT + chemotherapy	Xerostomia: RTOG Salivary gland hypofunction: NA		The prevalence of grade 2 or worse xerostomia was not significantly different in the experimental arm (8%) compared to the control arm (30%) (median duration of follow-up was 36 months)	Some concerns: randomization process, deviations from the intended interventions and measurement of the outcome
Nutting CM, Morden JP, Harrington KJ, Urbano TG, Bhide SA, Clark C, Miles EA, Miah AB, Newbold K, Tanay M, Adab F, Jefferies SJ, Scrase C, Yap BK, A’Hern RP, Sydenham MA, Emson M, & Hall E. (2011). Parotid-sparing intensity modulated versus conventional radiotherapy in head and neck cancer (PARSPORT): A phase 3 multicentre randomised controlled trial. The Lancet Oncology, 12(2), 127–136	94	Experimental arm: Parotid-sparing IMRT Control arm: Conventional radiation therapy	Xerostomia: LENT-SOMA Salivary gland hypofunction: uwsfr, spsfr		At 12 months the prevalence of grade 2 or worse xerostomia was significantly lower in the experimental arm (38%) compared to the control arm (74%) Selective stimulated contralateral parotid saliva flow rate was reported significantly higher in the experimental group (salivary flow rates not reported)	Some concerns: deviations from the intended interventions and selection of the reported results
Peng G, Wang T, Yang KY, Zhang S, Zhang T, Li Q, Han J, & Wu G. (2012). A prospective, randomized study comparing outcomes and toxicities of intensity-modulated radiotherapy vs. conventional two-dimensional radiotherapy for the treatment of nasopharyngeal carcinoma. Radiotherapy and Oncology, 104(3), 286–293	616	Experimental arm: IMRT Control arm: 2D-CRT	Xerostomia: CTCAE Salivary gland hypofunction: NA		Late (6 months or more after radiation therapy) the prevalence of grade 2 or worse xerostomia was significantly lower in the experimental arm (9.5%) compared to the control arm (29%)	Some concerns: deviations from the intended interventions and selection of the reported results
Songthong AP, Kannarunimit D, Chakkabat C, & Lertbutsayanukul C. (2015). A randomized phase II/III study of adverse events between sequential (SEQ) versus simultaneous integrated boost (SIB) intensity modulated radiation therapy (IMRT) in nasopharyngeal carcinoma; preliminary result on acute adverse events. Radiation Oncology, 10, 166	122	Experimental arm: Sequential phase IMRT Control arm: Simultaneous integrated boost IMRT	Xerostomia: CTCAE Salivary gland hypofunction: NA		Within 3 months after treatment completion, the prevalence of acute xerostomia was not significantly different in the experimental arm (9.6%) compared to the control arm (7.6%)	High
Tandon S, Gairola M, Ahlawat P, Rawat S, Aggarwal A, Sharma K, Tiwari S, Karimi AM, Muttagi V, Sachdeva N, & Bhushan M. (2018). Randomized controlled study comparing simultaneous modulated accelerated radiotherapy versus simultaneous integrated boost intensity modulated radiotherapy in the treatment of locally advanced head and neck cancer. Journal of the Egyptian National Cancer Institute, 30(3), 107–115	60	Experimental arm: Simultaneous integrated boost Control arm: Simultaneous modulated accelerated radiation therapy	Xerostomia: CTCAE Salivary gland hypofunction: NA		The prevalence of grade 3 or worse acute and late xerostomia (more than 2 years following treatment completion) was similar and not statistically significant between the two study groups (data provided only for acute toxicities where 70% of participants in the experimental arm versus 50% in the control arm suffered from grade 3 or worse xerostomia)	Low
Roel J.H.M. Steenbakkers, Maria I. van Rijn–Dekker, Monique A. Stokman, Roel G.J. Kierkels, Arjen van der Schaaf, Johanna G.M. van den Hoek, Hendrik P. Bijl, Maria C.A. Kramer, Robert P. Coppes, Johannes A. Langendijk, Peter van Luijk, Parotid Gland Stem Cell Sparing Radiation Therapy for Patients With Head and Neck Cancer: A Double-Blind Randomized Controlled Trial, International Journal of Radiation Oncology*Biology*Physics, 112 (2), 2022, 306–316,	102	Experimental arm: stem cell sparing Control arm: standard parotid-sparing	Xerostomia: individual item of the European Organization for Research and Treatment for Cancer Quality of Life Questionnaire Head and Neck (QLQ-H&N35), CTCAE Salivary gland hypofunction: spsfr		At 12 months, there was no difference between parotid gland salivary function or xerostomia between groups	Low
Preventative acupuncture						
Garcia MK, Meng Z, Rosenthal DI, Shen Y, Chambers M, Yang P, Wei Q, Hu C, Wu C, Bei W, Prinsloo S, Chiang J, Lopez G, & Cohen L. (2019). Effect of True and Sham Acupuncture on Radiation-Induced Xerostomia Among Patients With Head and Neck Cancer: A Randomized Clinical Trial. JAMA network open, 2(12), e1916910	339	Experimental arm: True acupuncture 3 days per week during a 6- to 7-week course of radiation therapy Control arm A: Sham-acupuncture using an acupuncture placebo device Control arm B: Standard of care (information about oral hygiene)	Xerostomia: Xerostomia Questionnaire Salivary gland hypofunction: Whole saliva flow rate (unclear if unstimulated or stimulated)		Significantly lower score indicating less xerostomia in the experimental arm compared to standard care 1 year after radiation therapy ended. (26.6 vs 34.8) No significant difference between true and sham acupuncture. Results varied based on center performing the intervention Results on whole saliva flow rate not reported	High
Meng Z, Kay Garcia M, Hu C, Chiang J, Chambers M, Rosenthal DI, Peng H, Wu C, Zhao Q, Zhao G, Liu L, Spelman A, Lynn Palmer J, Wei Q, & Cohen L. (2012). Sham-controlled, randomised, feasibility trial of acupuncture for prevention of radiation-induced xerostomia among patients with nasopharyngeal carcinoma. European Journal of Cancer, 48(11), 1692–1699	23	Experimental arm: Acupuncture 3 times a week during radiation therapy Control arm: Sham acupuncture	Xerostomia: Xerostomia Questionnaire Salivary gland hypofunction: uwsfr, swsfr		Xerostomia score in the acupuncture group was significantly lower than the control group up to 1 month following radiation therapy completion (week 11: 17.1 versus 38.9) There was no significant difference in uwsfr and swsfr	Some concerns: measurement of outcome domain
Meng Z, Garcia MK, Hu C, Chiang J, Chambers M, Rosenthal DI, Peng H, Zhang Y, Zhao Q, Zhao G, Liu L, Spelman A, Palmer JL, Wei Q, & Cohen L. (2012). Randomized controlled trial of acupuncture for prevention of radiation-induced xerostomia among patients with nasopharyngeal carcinoma. Cancer, 118(13), 3337–3344	86	Experimental arm: Acupuncture 3 days/week during radiation therapy Control arm: Standard of care	Xerostomia: Xerostomia Questionnaire Salivary gland hypofunction: uwsfr, swsfr		Xerostomia score in the acupuncture group was significantly lower than the control group up to 6 months following radiation therapy completion) (21.9 versus 34.0) Uwsfr and swsfr were significantly higher in the experimental arm at specific time points (salivary flow rates not reported)	Some concerns: deviation from the intended intervention and measurement of the outcome
Muscarinic agonist stimulation, bethanechol during radiation therapy						
Jaguar GC, Lima ENP, Kowalski LP, Pellizzon AC, Carvalho AL, Boccaletti KW, & Alves FA. (2015). Double blind randomized prospective trial of bethanechol in the prevention of radiation-induced salivary gland dysfunction in head and neck cancer patients. Radiotherapy and Oncology: Journal of the European Society for Therapeutic Radiology and Oncology, 115(2), 253–256	97	Experimental arm: Bethanechol tablet (25 mg) twice a day from the beginning of radiation therapy and continued until 1 month after the end of treatment Control arm: Placebo	Xerostomia: Xerostomia questionnaire Salivary gland hypofunction: uwsfr, swsfr		At 3 months, the prevalence of grade 2–3 xerostomia was significantly lower in the experimental group (38%) compared to placebo group (71%) Significantly higher uwsfr and swsfr at selected time point (after 2 months of the end of radiation therapy, the mean uwsfr and swsfr were 0.29 and 0.38 ml/min for the bethanechol group versus 0.06 and 0.23 ml/min for the placebo group)	Low
Campanhã D, PereiraLima EN, Alves FA & Jaguar GC. (2022). Bethanechol used to prevent salivary gland dysfunction in patients submitted to radioactive iodine therapy: A double-blind, placebo-controlled, randomized study. Journal of Stomatology, Oral and Maxillofacial Surgery, 123(5), e626-e630	41	Experimental arm: Bethanechol tablet (25 mg) twice a day beginning 2 h after iodine therapy and continued one month after the end of treatment Control arm: Placebo	Xerostomia: Xerostomia questionnaire Salivary gland hypofunction: uwsfr QoL: UW-QOL questionnaire		At 10 days, the prevalence of grade 2 xerostomia was significantly lower in the experimental group (0%) compared to the control group (4.3%) At 30 days, the prevalence of grade 2 xerostomia was significantly lower in the experimental group (0%) compared to control group (10%) At 3 months, results were not statistically significant No difference in salivary gland hypofunction	Low
Vitamin E and antioxidants						
Chung MK, Kim DH, Ahn YC, Choi JY, Kim EH, & Son Y-I. (2016). Randomized Trial of Vitamin C/E Complex for Prevention of Radiation-Induced Xerostomia in Patients with Head and Neck Cancer. Otolaryngology–Head and Neck Surgery: Official Journal of American Academy of Otolaryngology-Head and Neck Surgery, 155(3), 423–430	45	Experimental arm: 100 IU vitamin E and 500 mg of vitamin C twice a day during radiation therapy Control arm: Placebo	Xerostomia: Xerostomia questionnaire (unclear validation) Salivary gland hypofunction: Salivary scintigraphy (data not collected)		Experimental group showed significant greater improvement in xerostomia score at 6 months (Experimental arm 5.4 at 6 months versus 8.1 at 1 month; *p* = .02; Control arm at 6 months 7.0 versus 7.0 at one month; *p* = .06) No between-group analyses performed	Some concerns: deviation from the intended intervention, measurement of the outcome
Sayed R, El Wakeel L, Saad AS, Kelany M, & El-Hamamsy M. (2020). Pentoxifylline and vitamin E reduce the severity of radiotherapy-induced oral mucositis and dysphagia in head and neck cancer patients: a randomized, controlled study. Medical Oncology, 37(1)	60	Experimental arm: Pentoxifylline 400 mg oral tablets twice a day and vitamin E 1000 mg capsule daily during radiation therapy Control arm: Placebo	Xerostomia: CTCAE Salivary gland hypofunction: NA		Fewer patients in the experimental arm compared to the control arm developed xerostomia (20% versus 27%) at follow-up within 3 months from treatment completion (statistical significance not reported)	Some concerns deviation from the intended intervention, measurement of the outcome
Submandibular gland transfer						
Jha N, Seikaly H, Harris J, Williams D, Sultanem K, Hier M, Ghosh S, Black M, Butler J, Sutherland D, Kerr P, & Barnaby P. (2009). Phase III randomized study: Oral pilocarpine versus submandibular salivary gland transfer protocol for the management of radiation-induced xerostomia. Head and Neck, 31(2), 234–243	120	Experimental arm: Submandibular salivary gland transfer procedure Control arm: Pilocarpine during and for 3 months after radiation therapy	Xerostomia: NA Salivary gland hypofunction: uwsfr, swsfr		At 6 months, uwsfr and swsfr were significantly higher in the experimental group (median uwsfr was 0.04 ml/min for the experimental group and 0.01 ml/min in the pilocarpine group; median swsfr was 0.18 ml min and 0.05 ml/min respectively)	Some concerns: randomization process, deviation from the intended intervention and measurement of the outcome
Zhang X, Liu F, Lan X, Yu L, Wu W, Wu X, Xiao F, & Li S. (2014). Clinical observation of submandibular gland transfer for the prevention of xerostomia after radiotherapy for nasopharyngeal carcinoma: a prospective randomized controlled study of 32 cases. Radiation Oncology (London, England), 9, 62	65	Experimental arm: Submandibular gland transfer to submental region Control arm: No intervention	Xerostomia: RTOG, VAS Salivary gland hypofunction: uwsfr		At 3, 6, and 12 months, the prevalence of grade 2 or worse xerostomia was significantly lower in the experimental arm than in the control arm (at 6 six months 28% in the experimental arm versus 88% in the control arm) At 5 years, VAS score in the experimental group was significantly lower (3.7) than in the control group (5.8) Significantly higher unstimulated whole saliva secretion was seen in the experimental arm (1.19 g, 1.28 g, 1.39 g) compared to the control arm (0.58 g, 0.63 g, 0.66 g)	High
Amifostine						
Bardet E, Martin L, Calais G, Alfonsi M, Feham NE, Tuchais C, Boisselier P, Dessard-Diana B, Seng SH, Garaud P, Aupérin A, & Bourhis J. (2011). Subcutaneous compared with intravenous administration of amifostine in patients with head and neck cancer receiving radiotherapy: Final results of the GORTEC 2000–02 phase III randomized trial. Journal of Clinical Oncology, 29(2), 127–133	291	Experimental arm: Intravenous amifostine 200 mg/m^2^ daily Control arm: Subcutaneous amifostine 500 mg	Xerostomia: Patients’ self-assessed symptom, RTOG Salivary gland hypofunction: uwsfr, swsfr		No significant difference in uwsfr and swsfr or grade 2 or worse xerostomia between the two study groups at 12 months (18% for experimental arm versus 26% for the control arm)	Some concerns: deviation from the intended intervention and measurement of the outcome
Lee MG, Freeman AR, Roos DE, Milner AD, & Borg MF. (2019). Randomized double-blind trial of amifostine versus placebo for radiation-induced xerostomia in patients with head and neck cancer. Journal of Medical Imaging and Radiation Oncology, 63(1), 142–150	44	Experimental arm: Amifostine (200 mg/m^2^ iv) 5 times a week during radiotherapy Control arm: Placebo	Xerostomia: RTOG Salivary gland hypofunction: NA		The prevalence of xerostomia grade 2 or worse at 12 months was lower (66% versus 82%) in the experimental arm compared to the control arm but not statistically significant	Low
Low-level laser therapy						
Gonnelli FAS, Palma LF, Giordani AJ, Deboni ALS, Dias RS, Segreto RA, & Segreto HRC. (2016). Low-Level Laser for Mitigation of Low Salivary Flow Rate in Head and Neck Cancer Patients Undergoing Radiochemotherapy: A Prospective Longitudinal Study. Photomedicine and Laser Surgery, 34(8), 326–330	27	Experimental arm: Low-level laser therapy 3 times a week during radiation therapy Control arm: Clinical care	Xerostomia: NA Salivary gland hypofunction: uwsfr		Experimental arm showed significantly higher uwsfr up to 30 days after radiation therapy completion compared to control arm (0.28 ml/min versus 0.11 ml/min)	Some concerns: randomization process, deviation from intended interventions, missing outcome data, and selection of reported results
Oton-Leite AF, Elias LSA, Morais MO, Pinezi JCD, Leles CR, Silva MAGS, & Mendonca EF. (2013). Effect of low level laser therapy in the reduction of oral complications in patients with cancer of the head and neck submitted to radiotherapy. Special Care in Dentistry: Official Publication of the American Association of Hospital Dentists, the Academy of Dentistry for the Handicapped, and the American Society for Geriatric Dentistry, 33(6), 294–300	60	Experimental arm: InGaAlP laser daily for 5 days before each radiation therapy session Control arm: Sham laser	Xerostomia: NA Salivary gland hypofunction: uwsfr, swsfr		Uwsfr and swsfr were significantly higher in the experimental arm compared to the control arm. Uwsfr was 0.14 ml/min versus 0.02 ml/min in the control group. Swsfr was 0.40 ml/min for the experimental arm versus 0.04 ml/min for the control arm Unclear follow-up: Assessment was performed 1 week after starting radiation therapy, and at the fifteenth and thirtieth sessions of radiation therapy	Some concerns: missing outcome data and selection of the reported results
Louzeiro GC, Cherubini K, de Figueiredo MAZ, & Salum FG. (2020). Effect of photobiomodulation on salivary flow and composition, xerostomia and quality of life of patients during head and neck radiotherapy in short term follow-up: A randomized controlled clinical trial. Journal of Photochemistry and Photobiology B: Biology, 209, 111,933	21	Experimental arm: InGaAlP laser immediately after the first radiotherapy session three times a week, preferably on alternate days, until the last radiotherapy session Control arm: Sham laser	Xerostomia: VAS, Treatment-Emergent Symptom Scale Salivary gland hypofunction: uwsfr, swsfr QoL: UW-QOL		No difference between groups was noted in relation to salivary flow rate, xerostomia or quality of life (follow-up 2 months)	Low
Miscellaneous preventative interventions						
Ang KK, Zhang Q, Rosenthal DI, Nguyen-Tan PF, Sherman EJ, Weber RS, Galvin JM, Bonner JA, Harris J, El-Naggar AK, Gillison ML, Jordan RC, Konski AA, Thorstad WL, Trotti A, Beitler JJ, Garden AS, Spanos WJ, Yom SS, & Axelrod RS. (2014). Randomized phase III trial of concurrent accelerated radiation plus cisplatin with or without cetuximab for stage III to IV head and neck carcinoma: RTOG 0522. Journal of Clinical Oncology, 32(27), 2940–2950	630	Experimental arm: Concurrent accelerated radiation plus cisplatin Control arm: Concurrent accelerated radiation plus cisplatin with cetuximab	Xerostomia: RTOG Salivary gland hypofunction: NA	The prevalence of all grade late xerostomia (more than 90 days from start of radiation therapy) was not significantly different in the experimental arm compared to the control arm (75% respectively)		Low
Arbabi-kalati F, Arbabi-kalati F, Deghatipour M, & Moghaddam AA. (2012). Evaluation of the efficacy of zinc sulfate in the prevention of chemotherapy-induced mucositis: A double-blind randomized clinical trial. Archives of Iranian Medicine, 15(7), 413–417	50	Experimental arm: 220 mg zinc sulfate capsules during chemotherapy Control arm: Placebo	Xerostomia: LENT-SOMA Salivary gland hypofunction: NA	Xerostomia score were significantly lower in the experimental arm compared to the placebo at specific time points (week 4 to week 16)		Low
Bolouri AJ, Pakfetrat A, Tonkaboni A, Aledavood SA, Najafi MF, Delavarian Z, Shakeri MT, & Mohtashami A. (2015). Preventing and therapeutic effect of propolis in radiotherapy induced mucositis of head and neck cancers: A triple-blind, randomized, placebo-controlled trial. International Journal of Cancer Management, 8(5)	20	Experimental arm: 15 ml of water based extract of propolis mouthwash 3 times a day for 5 weeks Control arm: 15 ml placebo mouthwash	Xerostomia: 5 standardized questions Salivary gland hypofunction: NA	At 5 weeks there were no significant difference in xerostomia score between the two arms		Some concerns: deviation from the intended intervention, missing outcome data and high concerns with regards to measurement of the outcome
Cao SM, Yang Q, Guo L, Mai HQ, Mo HY, Cao KJ, Qian CN, Zhao C, Xiang YQ, Zhang XP, Lin ZX, Li WX, Liu Q, Qiu F, Sun R, Chen QY, Huang PY, Luo DH, Hua YJ, Wu YS, Lv X, Wang L, Xia WX, Tang LQ, Ye YF, Chen MY, Guo X, & Hong MH. (2017). Neoadjuvant chemotherapy followed by concurrent chemoradiotherapy versus concurrent chemoradiotherapy alone in locoregionally advanced nasopharyngeal carcinoma: A phase III multicentre randomised controlled trial. European Journal of Cancer, 75, 14–23	476	Experimental arm: Neoadjuvant (cisplatin and fluorouracil) followed by concurrent chemoradiotherapy Control arm: Concurrent chemoradiotherapy (80 mg/m^2^ cisplatin every 3 weeks)	Xerostomia: RTOG Salivary gland hypofunction: NA	The prevalence of acute grade 3 or worse xerostomia was higher in the experimental arm (2%) compared to the control arm (1%)		Some concerns: Randomization process, deviation from the intended intervention, missing outcome data and measurement of the outcome
Charalambous A, Lambrinou E, Katodritis N, Vomvas D, Raftopoulos V, Georgiou M, Paikousis L, & Charalambous M. (2017). The effectiveness of thyme honey for the management of treatment-induced xerostomia in head and neck cancer patients: A feasibility randomized control trial. European Journal of Oncology Nursing, 27, 1–8	72	Experimental arm: Thyme honey oral rinses (20 ml of thyme honey diluted in 100 ml of purified water) Control arm: Saline rinses	Xerostomia: Xerostomia Questionnaire, CTCAE Salivary gland hypofunction: NA	At 26 weeks, xerostomia scores were significantly lower in the experimental arm (0.22) compared to the control arm (1.28)		Low
Chen L, Hu CS, Chen XZ, Hu GQ, Cheng ZB, Sun Y, Li WX, Chen YY, Xie FY, Liang SB, Chen Y, Xu TT, Li B, Long GX, Wang SY, Zheng BM, Guo Y, Sun Y, Mao YP, Tang LL, Chen YM, Liu MZ, & Ma J. (2017). Adjuvant chemotherapy in patients with locoregionally advanced nasopharyngeal carcinoma: Long-term results of a phase 3 multicentre randomised controlled trial. European Journal of Cancer, 75, 150–158	251	Experimental arm: Concurrent (cisplatin) and adjuvant chemotherapy (cisplatin and fluorouracil) Control arm: Concurrent chemotherapy	Xerostomia: RTOG Salivary gland hypofunction: NA	The prevalence of grade 3–4 late xerostomia (the median follow-up was 68.4 months) was not significant different in the experimental arm (7%) compared to the control arm (6%)		High
Driessen CML, De Boer JP, Gelderblom H, Rasch CRN, De Jong MA, Verbist BM, Melchers WJG, Tesselaar MET, Van Der Graaf WTA, Kaanders JHAM, & Van Herpen CML. (2016). Induction chemotherapy with docetaxel/cisplatin/5-fluorouracil followed by randomization to two cisplatin-based concomitant chemoradiotherapy schedules in patients with locally advanced head and neck cancer (CONDOR study) (Dutch Head and Neck Society 08–01): A randomized phase II study. European Journal of Cancer, 52, 77–84	65	Experimental arm: Concurrent chemotherapy (cisplatin 40 mg/m2) + accelerated radiation therapy Control arm: Concomitant chemotherapy (cisplatin 100 mg/m2) + conventional radiation therapy	Xerostomia: RTOG Salivary gland hypofunction: NA	The prevalence of all grade xerostomia (unclear if acute or late, median follow-up of the study was 38 months) was lower in the experimental arm (35%) compared to control arm (50%). Statistical significance not reported		High
Fasanaro E, Del Bianco P, Groff E, Riva A, Petrangolini G, Busato F, Stritoni P, Scarzello G, Loreggian L, & De Salvo GL (2022). Role of SAMITAL in the Prevention and Treatment of Chemo-Radiotherapy-Induced Oral Mucositis in Head and Neck Carcinoma: A Phase 2, Randomized, Double-Blind, Placebo-Controlled Clinical Trial (ROSAM). Cancers, 14, 6192	116	Experimental arm: SAMITAL granules for oral suspension of 20 ml, four-time daily for 11 weeks Control arm: Placebo	Xerostomia: Xerostomia Questionnaire Salivary gland hypofunction: NA	Both the OMAS and the XQ significantly deteriorated over time, with no statistically significant difference between the two treatment arms		Low
Ghosh-Laskar S, Kalyani N, Gupta T, Budrukkar A, Murthy V, Sengar M, Chaukar D, Pai P, Chaturvedi P, D’Cruz A, & Agarwal J. (2016). Conventional radiotherapy versus concurrent chemoradiotherapy versus accelerated radiotherapy in locoregionally advanced carcinoma of head and neck: Results of a prospective randomized trial. Head and Neck, 38(2), 202–207	168	Experimental arm: Accelerated radiation therapy Control arm A: Concurrent chemotherapy Control arm B: Conventional radiation therapy	Xerostomia: RTOG Salivary gland hypofunction: NA	Xerostomia: 31% of patients in the experimental arm, 42% of patients in the control A arm and 23% patients in the control B arm developed late (1 to 5 years after randomization) grade 2–3 xerostomia Statistical significance not reported		Some concerns: randomization process, deviation from the intended intervention and measurement of the outcome
Giralt, Trigo J, Nuyts S, Ozsahin M, Skladowski K, Hatoum G, Daisne JF, Yunes Ancona AC, Cmelak A, Mesía R, Zhang A, Oliner KS, & VanderWalde A. (2015). Panitumumab plus radiotherapy versus chemoradiotherapy in patients with unresected, locally advanced squamous-cell carcinoma of the head and neck (CONCERT-2): A randomised, controlled, open-label phase 2 trial. The Lancet Oncology, 16(2), 221–232	151	Experimental arm: Radiation therapy and panitumumab (three cycles 9 mg/kg every 3 weeks) Control arm: Chemoradiotherapy (cisplatin)	Xerostomia: CTCAE Salivary gland hypofunction: NA	13% patients in the experimental group and 11% patients in the control group developed grade 2–3 xerostomia (median follow-up was 107.5 weeks for patients in the control arm and 123 weeks for patients in the control arm) Statistical significance not reported		Some concerns: randomization process, deviation from the intended intervention, and selection of the reported results
Giralt J, Tao Y, Kortmann RD, Zasadny X, Contreras-Martinez J, Ceruse P, Arias de la Vega F, Lalla RV, Ozsahin EM, Pajkos G, Mazar A, Attali P, Bossi P, Vasseur B, Sonis S, Henke M, & Bensadoun RJ. (2020). Randomized Phase 2 Trial of a Novel Clonidine Mucoadhesive Buccal Tablet for the Amelioration of Oral Mucositis in Patients Treated With Concomitant Chemoradiation Therapy for Head and Neck Cancer. International Journal of Radiation Oncology Biology Physics, 106(2), 320–328	183	Experimental arm: Daily local clonidine 50 µg mucobuccal tablet Control arm A: Daily local clonidine 100 µg mucobuccal tablet Control arm B: Placebo	Xerostomia: CTCAE Salivary gland hypofunction: NA	40% patients in the experimental arm, 26.6% patients in the control A, and 27.4% in the control B arm developed xerostomia during radiation. The difference was not statistically significant		Low
Gunn GB, Mendoza TR, Garden AS, Wang XS, Shi Q, Morrison WH, Frank SJ, Phan J, Fuller CD, Chambers MS, Hanna EY, Lu C, Rosenthal DI, & Cleeland CS. (2020). Minocycline for symptom reduction during radiation therapy for head and neck cancer: a randomized clinical trial. Supportive Care in Cancer, 28(1), 261–269	40	Experimental arm: Minocycline (100 mg twice daily) Control arm: Placebo during radiation therapy	Xerostomia: MDASI-HN Salivary gland hypofunction: NA	Six weeks after radiotherapy completion there were no significant difference in symptoms between the two arms		Some concerns: missing outcome data
Hakim A, Ghoshal S, Verma R, & Sharma SC. (2019). Comparison of Functional Organ Preservation by Concomitant Boost Radiotherapy Versus Concurrent Chemoradiation in Locally Advanced Carcinoma of Larynx or Hypopharynx: A Prospective Randomized Study. Indian Journal of Otolaryngology and Head and Neck Surgery, 71(3), 360–366	40	Experimental arm: Concomitant boost radiation therapy Control arm: Chemoradiation (cisplatin)	Xerostomia: CTCAE Salivary gland hypofunction: NA	15% of participant in the experimental arm and 55% of patients in the control arm developed late xerostomia of any grade		Some concerns: domain selection of the reported results
Heukelom J, Lopez-Yurda M, Balm AJM, Wijers OB, Buter J, Gregor T, Wiggenraad R, De Boer JP, Tan IB, Verheij M, Sonke JJ, & Rasch CR. (2016). Late follow-up of the randomized radiation and concomitant high-dose intra-arterial or intravenous cisplatin (RADPLAT) trial for advanced head and neck cancer. Head and Neck, 38, E488-E493	237	Experimental arm: Intra-arterial cisplatin-based chemoradiation Control arm: Intravenous cisplatin-based chemoradiation	Xerostomia: RTOG Salivary gland hypofunction: NA	There was no significant difference in the prevalence of late grade 3 or worse xerostomia between the two arms (median follow-up was 90 months)		Low
Kim JW, Kim MG, Lee HJ, Koh Y, Kwon JH, Kim I, Park S, Kim BK, Oh JM, Kim KI, & Yoon SS. (2017). Topical recombinant human epidermal growth factor for oral mucositis induced by intensive chemotherapy with hematopoietic stem cell transplantation: Final analysis of a randomized, double-blind, placebo-controlled, phase 2 trial. PLoS ONE, 12(1)	138	Experimental arm: Recombinant human epidermal growth factor oral spray (50 microg/ml) Control arm: Placebo	Xerostomia: CTCAE Salivary gland hypofunction: NA	There was no significant difference in the prevalence of acute grade 1 xerostomia between the two arms (no grade 3 or 4 xerostomia were noted in the study period)		Low
Le Q-T, Kim HE, Schneider CJ, Murakozy G, Skladowski K, Reinisch S, Chen Y, Hickey M, Mo M, Chen M-G, Berger D, Lizambri R, & Henke M. (2011). Palifermin reduces severe mucositis in definitive chemoradiotherapy of locally advanced head and neck cancer: a randomized, placebo-controlled study. Journal of Clinical Oncology: Official Journal of the American Society of Clinical Oncology, 29(20), 2808–2814	188	Experimental arm: Chemoradiotherapy with cisplatin and palifermin (180 µg/kg intravenous) for 7 weeks Control arm: Radiation therapy with cisplatin and intravenous placebo for 7 weeks	Xerostomia: RTOG Salivary gland hypofunction: NA	At 4 months after radiotherapy completion the prevalence of xerostomia grade 2 or worse was lower (67% versus 80%) in the experimental arm compared to the control arm but not statistically significant		Low
Mesía R, Henke M, Fortin A, Minn H, Yunes Ancona AC, Cmelak A, Markowitz AB, Hotte SJ, Singh S, Chan ATC, Merlano MC, Skladowski K, Zhang A, Oliner KS, VanderWalde A, & Giralt J. (2015). Chemoradiotherapy with or without panitumumab in patients with unresected, locally advanced squamous-cell carcinoma of the head and neck (CONCERT-1): A randomised, controlled, open-label phase 2 trial. The Lancet Oncology, 16(2), 208–220	153	Experimental arm: Panitumumab + chemoradiotherapy (three cycles of intravenous panitumumab 9·0 mg/kg every 3 weeks plus cisplatin 75 mg/m^2^) Control arm: Cisplatin-based chemoradiotherapy (three cycles of cisplatin 100 mg/m^2^)	Xerostomia: CTCAE Salivary gland hypofunction: NA	The prevalence of grade 3 or worse xerostomia was the same in the experimental arm and control arm (2%). The prevalence of grade 2 was 30% for the experimental group and 27% for the control group Median follow-up was 106 weeks for patients in the experimental arm and 110 weeks for patients in the control arm Statistical significance not reported		Low
Onseng K, Johns NP, Khuayjarernpanishk T, Subongkot S, Priprem A, Hurst C, & Johns J. (2017). Beneficial Effects of Adjuvant Melatonin in Minimizing Oral Mucositis Complications in Head and Neck Cancer Patients Receiving Concurrent Chemoradiation. Journal of Alternative and Complementary Medicine, 23(12), 957–963	39	Experimental arm: 20 mg melatonin gargle before each irradiation and 20 mg melatonin capsules daily during chemoradiation Control arm: Placebo	Xerostomia: CTCAE Salivary gland hypofunction: NA	At the end of the treatment (day 49) there were no significant differences in the prevalence of grade 2 or worse xerostomia between groups		Low
Paterson C, Thomson MC, Caldwell B, Young R, McLean A, Porteous S, Clark S, Messow CM, Kean S, Grose D, Lamb C, Rizwannullah M, James A, Schipani S, Wilson C, Rulach R, & Jones R. (2019). Radiotherapy-induced xerostomia: a randomised, double-blind, controlled trial of Visco-ease™ oral spray compared with placebo in patients with cancer of the head and neck. British Journal of Oral and Maxillofacial Surgery, 57(10), 1119–1125	43	Experimental arm: Viscosity-reducing mouth spray at least one spray twice a day during radiation therapy Control arm: 0.9% physiological saline	Xerostomia: Groningen radiotherapy-induced xerostomia questionnaire Salivary gland hypofunction: NA	At 6 weeks, there was no difference in xerostomia score between the two groups		Some concerns: deviation from intended intervention
Patil VM, Noronha V, Joshi A, Agarwal J, Ghosh-Laskar S, Budrukkar A, Murthy V, Gupta T, Mahimkar M, Juvekar S, Arya S, Mahajan A, Agarwal A, Purandare N, Rangarajan V, Balaji A, Chaudhari SV, Banavali S, Kannan S, Bhattacharjee A, D’Cruz AK, Chaturvedi P, Pai PS, Chaukar D, Pantvaidya G, Nair D, Nair S, Deshmukh A, Thiagarajan S, Mathrudev V, Manjrekar A, Dhumal S, Maske K, Bhelekar AS, Nawale K, Chandrasekharan A, Pande N, Goel A, Talreja V, Simha V, Srinivas S, Swami R, Vallathol DH, Dsouza H, Shrirangwar S, Turkar S, Abraham G, Thanky AH, Patel U, Pandey MK, & Prabhash K. (2019). A randomized phase 3 trial comparing nimotuzumab plus cisplatin chemoradiotherapy versus cisplatin chemoradiotherapy alone in locally advanced head and neck cancer. Cancer, 125(18), 3184–3197	536	Experimental arm: Nimotuzumab (200 mg) plus cisplatin-radiation arm Control arm: Cisplatin-radiation arm (30 mg/m^2^)	Xerostomia: CTCAE Salivary gland hypofunction: NA	The prevalence of late (> 90 days from treatment completion) all grade xerostomia was not significantly different in the experimental arm (96%) and control arm (98%)		Some concerns: measurement of the outcome
Pimenta Amaral TM, Campos CC, Moreira dos Santos TP, Leles CR, Teixeira AL, Teixeira MM, Bittencourt H, & Silva TA. (2012). Effect of salivary stimulation therapies on salivary flow and chemotherapy-induced mucositis: a preliminary study. Oral Surgery, Oral Medicine, Oral Pathology and Oral Radiology, 113(5), 628–637	35	Experimental arm: TENS 7 days before HSCT and up to 30 days after Three sessions weekly Control arm A: No intervention Control arm B: Mechanical sialagogues (chewing gum) 4 times a day for 10 min Control arm C: Combination of mechanical/electrical sialagogues	Xerostomia: NA Salivary gland hypofunction: uwsfr, swsfr	No significant differences in uwsfr and swsfr between the groups		High
Poddar J, Sharma AD, Kunikullaya SU, & Neema JP. (2017). Comparison of conventional fractionation (five fractions per week) and altered fractionation (six fractions per week) in stage I and II squamous cell carcinoma of oropharynx: An institutional study. Indian Journal of Cancer, 54(1), 6–10	60	Experimental arm: Accelerated 6 fractions radiation therapy per week Control arm: Conventional 5 fractions radiation therapy per week	Xerostomia: CTCAE Salivary gland hypofunction: NA	No significant difference in the prevalence of all grade xerostomia at 1 year between groups Note: 25 patients were available for analysis in both arms		High
Saad E, Radwan RH, & Hadi EA. (2020). Comparison between hypo-fractionated dose-escalated volumetric modulated arc therapy and conventional concurrent chemo-radiation in locally advanced head and neck cancer: A pilot study. Journal of Radiotherapy in Practice, 19(2), 132–138	63	Experimental arm: 70 Gy in 35 fractions in 7 weeks concurrently with cisplatin 100 mg/m^2^ every 3 weeks for 3 doses Control arm: 74 Gy in 33 fractions in 6·5 weeks	Xerostomia: CTCAE Salivary gland hypofunction: NA	The prevalence of late (> 90 days) all grade xerostomia was similar in the two groups (94%) with grade 3 or worse xerostomia significantly more prevalent in the experimental arm (48%) than control arm (13%)		High
Sharma, R., Vats, S., Seam, R., Gupta, M., Negi, R. R., Fotedar, V., & Singh, K. (2023). A Comparison of the Toxicities in Patients With Locally Advanced Head and Neck Cancers Treated With Concomitant Boost Radiotherapy Versus Conventional Chemoradiation. Cureus, 15(4), e38362	46	Experimental arm: Concomitant boost radiation therapy Control arm: Chemoradiation (cisplatin)	Xerostomia: 4’point Likert scale questionnaire Salivary gland hypofunction: NA	No significant difference in the prevalence of xerostomia between groups		Some concerns: domain selection of the reported results
Sio TT, Blanchard MJ, Novotny PJ, Patel SH, Rwigema JCM, Pederson LD, McGee LA, Gamez ME, Seeger GR, Martenson JA, Grover Y, Neben Wittich MA, Garces YI, Foote RL, Miller RC, & Halyard MY. (2019). N-Acetylcysteine Rinse for Thick Secretion and Mucositis of Head and Neck Chemoradiotherapy (Alliance MC13C2): A Double-Blind Randomized Clinical Trial. Mayo Clinic Proceedings, 94(9), 1814–1824	32	Experimental arm: 10% N-Acetylcysteine oral rinse (2500 mg daily) 5 times daily during radiation therapy and 2 weeks after Control arm: Placebo oral rinse	Xerostomia: Groningen Radiotherapy-Induced Xerostomia questionnaire Salivary gland hypofunction: NA	Xerostomia score during radiation therapy and up to 3 months upon treatment completion were significantly lower in the experimental arm compared to control arm (27.3 versus 44.1). Results were not sustained after 3 months		Low
Tallari RV, Singh OP, Yogi V, & Yadav S. (2017). Five versus ten fractions per week radiotherapy in locally advanced head and neck cancer. Journal of Cancer Research and Therapeutics, 13(2), 224–229	100	Experimental arm: Hyperfractionated radiation therapy (10 sessions per week) Control arm: Conventional fractionated radiation therapy (5 sessions per week)	Xerostomia: RTOG Salivary gland hypofunction: NA	The difference in the prevalence of late (> 90 days) grade 2 or worse xerostomia was not significantly different in the experimental arm (23%) and control arm (29%)		High
Tang LQ, Chen DP, Guo L, Mo HY, Huang Y, Guo SS, Qi B, Tang QN, Wang P, Li XY, Li JB, Liu Q, Gao YH, Xie FY, Liu LT, Li Y, Liu SL, Xie HJ, Liang YJ, Sun XS, Yan JJ, Wu YS, Luo DH, Huang PY, Xiang YQ, Sun R, Chen MY, Lv X, Wang L, Xia WX, Zhao C, Cao KJ, Qian CN, Guo X, Hong MH, Nie ZQ, Chen QY, & Mai HQ. (2018). Concurrent chemoradiotherapy with nedaplatin versus cisplatin in stage II–IVB nasopharyngeal carcinoma: an open-label, non-inferiority, randomised phase 3 trial. The Lancet Oncology, 19(4), 461–473	402	Experimental arm: Nedaplatin 100 mg/m^2^ on days 1, 22, and 43 for three cycles with IMRT Control arm: Cisplatin 100 mg/m^2^ on days 1, 22, and 43 for three cycles with IMRT	Xerostomia: CTCAE Salivary gland hypofunction: NA	The difference in the prevalence of any grade late xerostomia was equal in the experimental and control arm (median follow-up was 47 months)		Some concerns: deviation from the intended intervention and measurement of the outcome
Tavakoli Ardakani M, Ghassemi S, Mehdizadeh M, Mojab F, Salamzadeh J, Ghassemi S, & Hajifathali A. (2016). Evaluating the effect of Matricaria recutita and Mentha piperita herbal mouthwash on management of oral mucositis in patients undergoing hematopoietic stem cell transplantation: A randomized, double blind, placebo controlled clinical trial. Complementary Therapies in Medicine, 29, 29–34	60	Experimental arm: *Matricaria recutita* and *Mentha piperita* herbal mouthwash three times a day for at least 30 s during radiation therapy Control arm: Placebo	Xerostomia: Unclear Salivary gland hypofunction: NA	“Dryness of oral cavity” was significantly lower in the experimental arm compared to the control arm (1.33 versus 2.29) No follow-up, the treatment was continued until the complete healing of oral mucositis or hospital discharge		Some concerns: all domains
Wang C, Wang P, Ouyang H, Wang J, Sun L, Li Y, Liu D, Jiang Z, Wang B, & Pan Z. (2018). Efficacy of Traditional Chinese Medicine in Treatment and Prophylaxis of Radiation-Induced Oral Mucositis in Patients Receiving Radiotherapy: A Randomized Controlled Trial. Integrative Cancer Therapies, 17(2), 444–450	70	Experimental arm: Traditional Chinese medicine spray (rhubarb, licorice, mint, *Scutellaria*, radix liriopes, red peony root, lumbricus, radix scrophulariae and forsythia) three times a day during radiation therapy Control arm: Recombinant human epidermal growth factor spray	Xerostomia: VAS Salivary gland hypofunction: NA	Xerostomia score was significantly different at each observation (weekly for 7 weeks of radiation) in favor of the experimental arm (VAS score at 7 weeks was 3.2 in the treatment group versus 5.86 in the control group)		High

*2D-CRT*, two-dimensional conventional radiation therapy; *3D-CRT*, three-dimensional conformal radiation therapy; *CTCAE*, Common Terminology Criteria for Adverse Events; *HSCT*, hematopoietic stem cell transplantation; *IMRT*, intensity-modulated radiation therapy; *LENT-SOMA*, Late Effects Normal Tissues-Subjective, Objective, Management, Analytic; *NA*, not available; *RTOG*, Toxicity criteria of the Radiation Therapy Oncology Group; *spsfr*, stimulated parotid saliva flow rate; *swsfr*, stimulated whole saliva flow rate; *QoL*, quality of life; *uwsfr*, unstimulated whole saliva flow rate; *VAS*, Visual Analogue Scale; *TENS*, transcutaneous electrical nerve stimulation

The majority of the studies focused on subjective outcome measures, i.e., the RTOG (Radiation Therapy Oncology Group)-xerostomia scoring criteria: 14, LENT-SOMA (Late Effects Normal Tissue Task Force—Subjective, Objective, Management, Analytic) scale: 2, CTCAE (Common Terminology Criteria for Adverse Events) grading criteria: 16, Xerostomia questionnaire: 10; MDASI – HN (M.D. Anderson Symptom Inventory—Head and Neck) module: 1, the Groningen Radiotherapy-Induced Xerostomia questionnaire: 2, VAS (Visual Analogue Scale): 3. Objective outcome measures used included unstimulated whole salivary flow rate: 13 (in one case it was unclear whether whole salivary flow rate was stimulated or not), stimulated whole salivary flow rate: 8, stimulated parotid flow rate: 2, scintigraphy: 1. Two studies included Quality of Life outcome measures using the EORTC (European Organisation for Research and Treatment of Cancer) QLQ-H&N35 (H&N35) in 1 study and The University of Washington Quality of Life Questionnaire in 1 study.

Overall, 11 studies were judged to be at high risk of bias, 17 at low risk of bias, and 23 were identified as having some concern with regard to the risk of bias according to the Cochrane risk-of-bias tool for randomized trials (RoB version 2). There were no RCT on checkpoint inhibitors and other biologicals.

### Types of preventive interventions

#### Intensity-modulated radiation therapy (IMRT)

Eight RCTs were identified that assessed IMRT and its effect on salivary gland function. All of the included studies addressed xerostomia [17, 22, 31, 45, 50, 58, 60, 62], and two studies addressed both xerostomia and salivary gland hypofunction [45, 60]. Description of individual studies is shown in Table 1 demonstrating significant lower prevalence of grade 2 or worse xerostomia in an experimental arm (IMRT) compared to control arms (either 3D conventional RT or 2D conventional RT), with follow-up ranging from 4 weeks to 5 years. There was no statistically significant difference in prevalence of xerostomia between IMRT and chemotherapy versus IMRT and recombinant human endostatin [31], between sequential versus simultaneous integrated boost IMRT [58] and with the use of simultaneous modulated accelerated radiotherapy (SMART) [62]. With regards to salivary gland hypofunction, it was documented that elective stimulated contralateral parotid saliva flow rate was significantly higher in an experimental IMRT group compared to 2D conventional RT [45]; however, the magnitude of effect was unclear as salivary flow rates were not reported in the manuscript. No significantly enhanced parotid function was observed in a more recent RCT when standard parotid-sparing was compared to stem cell-sparing techniques [60].

#### Preventive acupuncture

Three RCTs assessed the effectiveness of preventive acupuncture to reduce the risk of developing salivary gland hypofunction and xerostomia during cancer therapies. All studies addressed xerostomia and salivary gland function [15, 37, 38], although in one study the results of salivary gland function were not reported [15]. Description of individual studies is shown in Table 1 demonstrating significant lower xerostomia score with the use of acupuncture compared to standard care (information about oral hygiene) with no statistically significant difference between true acupuncture and sham acupuncture. Follow-up was between one month and six months.

#### Bethanechol

Two studies on muscarinic agonist stimulants (bethanecol) performed in the same institution were included in this systematic review, one involving head and neck cancer patients [25] and a more recent one involving patients undergoing radioactive iodine therapy [6]. Both assessed xerostomia and salivary gland function. In the first clinical trial, bethanechol was associated with lower prevalence of xerostomia compared to the placebo group and significantly higher unstimulated and stimulated whole salivary flow rates at selected time points [25]. In the second study, the benefits on xerostomia were maintained ten days after treatment was commenced and up to 1 month after the end of the treatment. There was no statistically significant difference in unstimulated salivary flow rate between groups [6].

#### Vitamin E and antioxidants

Two studies assessed the preventive effect of vitamin E and antioxidants on xerostomia during radiation therapy [10, 54].

There is unclear evidence of the preventive efficacy of vitamin C/E complex supplementation due to unclear validity of study questionnaire and data analysis (between-group analyses not performed) [10] and unclear magnitude of effect with regards to the potential benefit of pentoxifylline and vitamin E compared to radiotherapy alone [54] as statistical significance was not reported in the study. Salivary gland hypofunction was assessed via scintigraphy; however, results were not reported in the manuscript.

#### Submandibular gland transfer

Two RCTs tested the feasibility of surgical transfer of one submandibular gland to the submental space (outside the radiation portal) [30, 69]. One of the studies assessed both salivary gland hypofunction and xerostomia [69], while the other study only addressed salivary gland hypofunction [30].

In one study, 120 participants were randomized to submandibular salivary gland transfer versus pilocarpine during and for three months after radiotherapy [30]. Although the study did not include a xerostomia-related outcome measure, at 6 months unstimulated and stimulated salivary function was higher in the experimental group compared to the pilocarpine group. A second study compared submandibular salivary gland transfer to the submental region versus no active intervention [69] showing lower prevalence of moderate to severe xerostomia in the experimental arm and higher unstimulated salivary flow than in the control arm up to 12 months following surgical treatment.

#### Amifostine

The effect of amifostine was assessed in two RCTs [3, 34]. One study assessed both salivary gland hypofunction and xerostomia [3], while the other study only assessed xerostomia [34].

Compliance with and efficacy of intravenous (IV) and subcutaneous (SC) amifostine was compared showing that the incidence of grade 2 or greater xerostomia was similar in both groups. There was no significant difference in unstimulated or stimulated salivary flow rates [3].

Another study randomized patients to receive intravenous amifostine or placebo prior to standard radiotherapy [34] showing that the prevalence of xerostomia grade 2 or greater at 12 months was not statistically different between groups. Of note, the trial closed prematurely after recruiting 44 of the intended 200 participants after three years.

#### Photobiomodulation therapy

Three RCTs were identified assessing the effect of photobiomodulation therapy on salivary gland function during radiation therapy [20, 36, 47]. Two studies addressed salivary gland hypofunction [20, 47] with one study addressing xerostomia, salivary gland hypofunction, and quality of life [36].

A RCT involved 27 patients undergoing head and neck radiotherapy randomised to receive either photobiomodulation therapy or standard clinical care [20]. The experimental arm showed higher unstimulated whole saliva flow rate up to 30 days after radiation therapy compared to the control arm but these results were not confirmed at 90 days. Similar results were obtained in a study that randomised participants into a laser or sham laser group [47]. Of note, this study has an unclear follow-up duration and unclear methods with regards to use of the intervention. A more recent study has shown no difference in xerostomia, salivary gland hypofunction, and quality of life up to 2 months following radiotherapy completion between laser and sham therapy [36].

#### Miscellaneous preventive interventions

Twenty-nine RCTs were identified assessing interventions that were categorized as miscellaneous, e.g., single testing of a broad variety of interventions, i.e., zinc sulfate capsules [2], propolis oral rinse [4], botanic drug extract (SAMITAL) [14], thyme honey oral rinse [8], topical clonidine [18], minocycline peroral tablets [21], melatonin oral rinse and capsules [46], viscosity-reducing oral spray [48], matricaria recutita and mentha piperita herbal mouthwash [64], traditional Chinese medicine spray (rhubarb, licorice, mint, scutellaria, radix liriopes, red peony root, lumbricus, radix scrophulariae and forsythia) [66], recombinant human epidermal growth factor oral spray [32], N-acetylcysteine oral rinse [57], TENS (transcutaneous electrical nerve stimulation) [51], palifermin [33], cetuximab [1], panitumumab [19, 41], nimotuzumab [49], nedaplatin [63], neoadjuvant or adjuvant cisplatin and fluorouracil [7, 9, 13], intra-arterial cisplatin [24], accelerated radiation therapy [16], boost radiation therapy [23, 56], and hyper- or hypofractionated radiation therapy [52, 53, 61].

Details of the included studies and quality assessments are provided in Table 1.

Zinc sulfate supplements compared with placebo capsules showed lower xerostomia score using the LENT-SOMA criteria only at specific time points (week 4 to week 16 and week 20) [2].

No statistically significant differences in xerostomia scores were observed following treatment with propolis—based oral rinse [4] or botanic drug extract (SAMITAL) granules [14], whereas the use of thyme honey oral rinse led to lower xerostomia score compared to the saline rinse control arm at the end of the treatment on week seven which was maintaned to week 26 [8].

With regards to altered radiotherapy regimens and chemotherapy regimen, no differences were observed in xerostomia score assessed via RTOG following concurrent accelerated radiation plus cisplatin when cetuximab was added to the treatment plan. Similarly, concurrent and adjuvant chemotherapy (cisplatin 80 mg/m^2^ and fluorouracil 800 mg/m^2^) did not provide benefit in terms of xerostomia compared with concurrent chemoradiotherapy alone [9] and when cisplatin—based chemoradiotherapy was compared to a combination of panitumumab and radiotherapy [19, 41]. The addition of nimotuzumab to cisplatin—based chemoradiotherapy did not lead to significantly difference in the prevalence of late xerostomia [49].

Neoadjuvant chemotherapy (cisplatin and fluorouracil) followed by concurrent chemoradiotherapy versus concurrent chemoradiotherapy (cisplatin every 3 weeks) alone led to a higher prevance of grade 3 or worse xerostomia (RTOG criteria) [7]. Of note, the authors have since published the long-term results with a 5-year follow-up confirming these results [67].

A study of patients with locally advanced head and neck cancer undergoing chemotherapy (docetaxel/cisplatin/5-fluorouracil) followed by randomization to either cisplatin with conventional radiotherapy or cisplatin with accelerated radiotherapy was prematurely terminated due to data safety monitoring; it is unclear in this study whether reported results were statistically significant [13]. Similarly, it is unclear whether results on a randomised trial comparing conventional radiotherapy to concurrent chemoradiotherapy or accelerated radiotherapy were statistically significant [16].

The use of a topical vasodilator (clonidine) delivered with a mucobuccal tablet showed that more patients developed xerostomia (CTCAE) during radiotherapy compared to placebo [18]. Of note, although participants were followed up every six months until two years after last visit, the data on adverse effects were collected only during the radiotherapy. Similarly, no benefit in xerostomia was detected following preventive use of systemic minocycline during radiotherapy [21].

For advanced (stage III/stage IVa) laryngeal and hypopharyngeal cancer, accellerated radiotherapy showed significantly lower prevalence of late xerostomia of any grade compared to concomitant conventional chemoradiotherapy [23]. When the same group of research recently performed a similar study on advanced head and neck cancer, the difference between groups was not significant [56].

With regards to inoperable squamous cell carcinoma of the oropharynx, oral cavity, or hypopharynx, there was no difference between participants assigned to high-dose intra-arterial or intra-venous cisplatin-based chemoradiation in the prevalence of late grade 3 or worse xerostomia between the two arms [24]. Similarly, there was no benefit in the prevalence of xerostomia following the use of recombinant human epidermal growth factor oral spray versus placebo [32], intravenous palifermin during chemoradiotherapy with cisplastin [33], melatonin gargle combined with metatonin capsules versus placebo [46], a viscosity-reducing mouth spray versus an equivalent physiological saline solution [48], and N-acetylcysteine rinse versus a placebo rinse after 3 months [57].

The effect of salivary stimulation therapies on salivary flow rate was assessed in patients receiving allogeneic HSCT randomised into four groups: control, mechanical sialogogue, transcutaneous electrical nerve stimulation (TENS) sialogogue, and combined mechanical/electrical sialogogue. Patient-reported outome measures were not collected in this study whose results showed no significant difference in unstimulated and stimulated whole salivary flow rates between the groups [51].

No significant difference in terms of acute and late radiation toxicity assessed with CTCAE criteria were reported in a study comparing conventional versus accelerated radiotherapy without chemotherapy [52].

A new radiotherapy protocol for patients not eligible to receive the standard concurrent chemo-radiation therapy by using a slightly dose-escalated accelerated hypofractionated regimen showed that the prevalence of late (> 90 days) all grade xerostomia was similar in the two groups (94%) with grade 3 or worse xerostomia significantly more prevalent in the experimental arm than control arm [53].

A study compared conventional five fractions per week radiotherapy with hyperfractionated radiotherapy (ten fractions per week) following induction chemotherapy [61] showing no difference in prevalence of late (> 90 days) grade 2 or worse xerostomia between groups.

Nedaplatin-based concurrent chemoradiotherapy regimen in patients with advanced nasopharyngeal carcinoma showing no difference compared to cisplatin-based chemotherapy in the prevalence of all grades late xerostomia between the two groups [63].

There is no clear benefit from the use of herbal mouthwash (*Matricaria recutita* and *Mentha piperita*) as the outcome measures of the study were unclear (“dryness of oral cavity”) [64].

There is some limited effect on the use of a traditional Chinese medicine (Chining decoction, CHIN) [66] when compared to a recombinant human epidermal growth factor spray in terms of acute xerostomia (VAS assessed at 7 weeks).

## Discussion

This systematic review evaluates the literature appearing since the MASCC/ISOO 2010 systematic review on approaches to prevent salivary gland hypofunction and xerostomia in patients undergoing nonsurgical cancer therapies and highlights the continued clinical importance of improving current preventive methods and development of new preventive strategies. In the time following publication of the guidelines [40], multiple approaches have been investigated to lower the radiation dose to the parotid glands and other organs at risk (OAR) compared with IMRT, including volumetric modulated arc therapy (VMAT), adaptive radiation therapy, intensity-modulated proton therapy, and spot-scanning proton arc. A double-blind RCT has been included in the present systematic review testing parotid gland stem cell-sparing radiation therapy showing no statistically significant better parotid function [60].

Since the publication of the ISOO/MASCC/ASCO 2021 guidelines on preventive strategies based on the present systematic review of the literature, recently published studies have been added to this [6, 14, 36] which have not changed the overall recommendations and suggestions.

Tissue-sparing radiation techniques and their effect on salivary gland function were addressed in eight RCTs, which showed high quality of evidence regarding the superiority of IMRT over conventional techniques in reducing moderate to severe acute and late xerostomia. Intensity-modulated proton therapy (IMPT) is a mode of proton delivery that offers the opportunity of reducing toxicities and improving patient quality of life; however, its utilization has been previously restricted by its costly expenses and limited availability [42]. There have been no published RCT so far. However, there are several ongoing clinical trials investigating IMPT in head and neck cancer (NCT01893307, ISRCTN: 16424014). A recent retrospective study has shown significant differences in terms of acute xerostomia of grade 2 or greater in favor of IMPT compared to IMRT [68]. Results from the ongoing clinical trials are awaited to confirm these findings and optimize patient selection.

Preventive acupuncture during radiation therapy was addressed in three RCTs [15, 37, 38], which suggested that this intervention may be considered to reduce xerostomia. However, the results did not show that acupuncture during radiation therapy will prevent salivary gland hypofunction, and the studies were assessed to raise some concern and high concern for risk of bias. This aligns with another systematic review published on the same research question [44].

Muscarinic agonist stimulation with bethanechol during radiation therapy suggested a preventive effect on salivary gland flow rate and xerostomia; however, the evidence is currently limited to two studies with low concern for risk of bias [25]. The previous systematic review has provided recommendations on the use of pilocarpine during radiotherapy [28] and no new studies were found in the present work.

A new study has been added on the use of bethanecol to prevent salivary gland hypofunction in patients undergoing radioactive iodine therapy showing short-term benefits in terms of reduction of the prevalence of xerostomia and impact on patients’ quality of life [6]. Further studies with longer follow-up would be welcomed on the efficacy of cholinergic parasympathomimetics.

Submandibular gland transfer was addressed in two RCTs which suggested that in clinically appropriate cases and when 2D and 3D radiation therapy is used, submandibular gland transfer could be used to prevent salivary gland hypofunction and xerostomia, although the studies raised some concern and high concern for risk of bias [30, 69]. There are no data on the use of submandibular gland transfer with contemporary tissue-sparing radiation modalities.

Amifostine was assessed in two RCTs that did not demonstrate a preventive effect on salivary gland hypofunction or xerostomia and the studies raised some concern and low concern for risk of bias [3, 34]. There is no evidence on the interaction and efficacy with newer parotid-sparing radiation modalities.

Photobiomodulation laser therapy as a preventive intervention for salivary gland hypofunction during radiation therapy was addressed in three RCTs [20, 36, 47] which showed no difference between groups in relation to salivary flow rate, xerostomia, or quality of life [36].

No conclusions could be drawn based on the RCTs on the included miscellaneous interventions due to scarcity of evidence and varying degrees of concern for risk of bias.

### Limitation

In the present review, the majority of studies raised some concerns of bias or were assessed to have high risk of bias. Some of the studies did not include patient-reported outcomes and had short follow-up. Given the chronic nature of the symptoms and impact on quality of life, this must be considered when applying the findings of this review in clinical practice.

Regarding evaluation of salivary gland function, discrepancies in the definitions of salivary gland hypofunction and xerostomia were observed. Considerable variation was found regarding clinician and patient-reported outcome measures. Furthermore, assessments were carried out at a wide range of time points during and after cancer treatment and studies lacked reporting on confounding factors known to influence salivary gland function, e.g., comorbidities and medication intake (xerogenic medications and polypharmacy).

## Recommendations and suggestions

Salivary gland hypofunction and xerostomia continue to be frequent and important complications in patients undergoing non-surgical cancer therapies, affecting oral functions, nutrition, and quality of life. This systematic review continues to support the potential of tissue-sparing radiation tecniques and IMRT to preserve salivary gland function in patients with in head and neck cancer, with limited evidence on other preventive strategies, including acupuncture and bethanecol. Heightened awareness and improved characterization of immune-related adverse events and biological therapies are required to develop evidence-based recommendations.

## Supplementary Information

Below is the link to the electronic supplementary material.

Supplementary file1 (DOCX 19 KB)
