# Correction to: A systematic review of salivary gland hypofunction and/or xerostomia induced by non‑surgical cancer therapies: prevention strategies

**DOI:** 10.1007/s00520-025-09327-7

**Published:** 2025-03-20

**Authors:** Valeria Mercadante, Derek K. Smith, Ragda Abdalla‑Aslan, Ana Andabak‑Rogulj, Michael T. Brennan, Graziella Chagas Jaguar, Haley Clark, Eduardo Rodrigues Fregnani, Luiz Alcino Gueiros, Allan Hovan, Seema Kurup, Alexa M. G. A. Laheij, Charlotte Duch Lynggaard, Joel J. Napeñas, Douglas E. Peterson, Sharon Elad, Stephanie Van Leeuwen, Arjan Vissink, Jonn Wu, Deborah P. Saunders, Siri Beier Jensen

**Affiliations:** 1 Eastman Dental Institute, University College London, London, England; 2 Vanderbilt University, Nashville, TN USA; 3 Bruce Rappaport Faculty of Medicine, Technion-Israel Institute of Technology, Haifa, Israel; 4 Department of Oral and Maxillofacial Surgery, Rambam Health Care Campus, Haifa, Israel; 5 Department of Oral Medicine, University of Zagreb School of Dental Medicine, Zagreb, Croatia; 6 Department of Oral Medicine/Oral & Maxillofacial Surgery, Atrium Health Carolinas Medical Center, Charlotte, NC USA; 7 Stomatology Department - A.C. Camargo Cancer Center, Sao Paulo, Brazil; 8 Department of Medical Physics, BC Cancer, Surrey, BC Canada; 9 Centro de Oncologia Molecular, Hospital Sírio-Libanês, São Paulo, Brazil; 10 Departamento de Clínica E Odontologia Preventiva, Universidade Federal de Pernambuco, Recife, Brazil; 11 Oral Oncology/Dentistry, British Columbia Cancer Agency-Vancouver Centre, Vancouver, BC Canada; 12 Department of Oral & Maxillofacial Diagnostic Sciences, School of Dental Medicine, Uconn Health, Farmington, CT USA; 13 Department of Oral Medicine, Academic Centre for Dentistry Amsterdam, University of Amsterdam and VU University, Amsterdam, The Netherlands; 14 Department of Oral and Maxillofacial Surgery, Amsterdam UMC, University of Amsterdam, Amsterdam, The Netherlands; 15 Department of Otorhinolaryngology, Head and Neck Surgery and Audiology, Rigshospitalet, Copenhagen University Hospital, Copenhagen, Denmark; 16 School of Dental Medicine and Neag Comprehensive Cancer Center, Uconn Health, Farmington, CT USA; 17 Eastman Institute for Oral Health, University of Rochester Medical Center, Rochester, NY USA; 18 Department of Dentistry, Radboud University Medical Center, Nijmegen, The Netherlands; 19 Department of Oral & Maxillofacial Surgery, University Medical Center Groningen, University of Groningen, Groningen, The Netherlands; 20 British Columbia Cancer Agency, University of British Columbia, Vancouver, Canada; 21 North East Cancer Center, Health Sciences North, Northern Ontario School of Medicine, Sudbury, ON Canada; 22 Department of Dentistry and Oral Health, Faculty of Health, Aarhus University, Aarhus, Denmark

**Correction to: Supportive Care in Cancer (2025) 33:87**

**https://doi.org/10.1007/s00520-024–09113-x**

The correct name should be Eduardo Rodrigues Fregnani and not Eduardo Fregnani, the citation should be Fregnani, ER.

The original article has been corrected.

