# Supplementary material for: A systematic review of salivary gland hypofunction and/or xerostomia induced by non-surgical cancer therapies: prevention strategies
Source: Support Care Cancer. 2025 Jan 10;33(2):87. doi: 10.1007/s00520-024-09113-x (PMC11723892; doi:10.1007/s00520-024-09113-x)
Supplement: Supplementary file 1 — Supplementary file1 (DOCX 19 KB) [file 520_2024_9113_MOESM1_ESM.docx]

Supplementary Document

Search Strategy

[Saliva] OR [Salivary Glands] OR [Salivation] OR [Salivary Gland Diseases] OR [Xerostomia] AND [Neoplasms] OR [Head and Neck Neoplasms/Radiotherapy] OR [Radiotherapy] OR [Anti-neoplastic Agents] OR [Antineoplastic Combined Chemo-therapy Protocols] OR [Combined Modality Therapy] OR [Total Body Irradiation] OR [Bone Marrow Transplantation] OR [Hematopoietic Stem Cell Transplantation] OR [Radioactive Iodine] OR [Biological Therapy] OR [Chemotherapy] OR [Immunotherapy] OR [Targeted Therapy] AND [Humans] AND [2009/01/01:2024/02/16].
